# Impaired Growth during Childhood in Patients with Primary Ciliary Dyskinesia

**DOI:** 10.1155/2013/731423

**Published:** 2013-12-12

**Authors:** Tamara Svobodová, Jana Djakow, Daniela Zemková, Adam Cipra, Petr Pohunek, Jan Lebl

**Affiliations:** ^1^Department of Pediatrics, 2nd Faculty of Medicine, University Hospital Motol, Charles University, 150 06 Prague, Czech Republic; ^2^Department of Pediatrics, Masaryk's Hospital, 400 11 Usti nad Labem, Czech Republic

## Abstract

Primary ciliary dyskinesia (PCD) leads to recurrent/chronic respiratory infections, resulting in chronic inflammation and potentially in chronic pulmonary disease with bronchiectasis. We analyzed longitudinal data on body length/height and body mass index (BMI) for 29 children and young adults with PCD aging 1.5–24 years (median, 14.5) who had been diagnosed at the age of 0.5–17 years (median, 8). Of these, 10 carried pathogenic mutations in either *DNAH5* or *DNAI1*. In children with PCD, body length/height progressively decreased from +0.40 ± 0.24 SDS (the 1st birthday), +0.16 ± 0.23 SDS (3 years old), and −0.13 ± 0.21 SDS (5 years old) to −0.54 ± 0.19 SDS (7 years old; *P* = 0.01 versus 0), −0.67 ± 0.21 SDS (9 years old; *P* = 0.005 versus 0), −0.52 ± 0.24 SDS (11 years old; *P* = 0.04 versus 0), and −0.53 ± 0.23 SDS (13 years old; *P* = 0.03 versus 0). These results reflect low growth rates during the childhood growth period. Thereafter, heights stabilized up to the age of 17 years. The growth deterioration was not dependent on sex or disease severity but was more pronounced in *DNAH5* or *DNAI1* mutation carriers. BMI did not differ from population standards, which suggests that nutritional deficits are not the cause of growth delay. We conclude that PCD leads to chronic deprivation with significant growth deterioration during childhood.

## 1. Introduction

Primary ciliary dyskinesia (PCD) is a genetically heterogeneous, autosomal-recessive disorder, characterized by structural and/or functional ciliary abnormalities with inefficient mucociliary clearance, resulting in recurrent or chronic infections of the upper and lower respiratory tract. PCD affects approximately 1 : 15,000–1 : 30,000 children [1, 2]. The main characteristics of PCD are recurrent sinopulmonary infections and decreased fertility. Situs viscerum inversus occurs in approximately 50% of patients. Symptoms frequently start during the neonatal period, and they include chronic rhinitis and a wet cough, with the gradual development of lower respiratory tract infections. These infections can lead to recurrent pneumonia, chronic bronchitis [3], and possibly bronchiectasis and progressive deterioration of lung function [4], requiring lung transplantation in the most severely affected subjects [5, 6]. The general well-being of children with PCD can be compromised by the activation of mediators of inflammation during chronic suppurative airway diseases, complicated during later stages by impaired lung function, by dyspnea-induced increased energy consumption, and by chronic hypoxia [7]. Both long-term activation of inflammation and chronic pulmonary disease, with dyspnea and hypoxia, have been shown to decrease IGF-I levels and to compromise children's growth, and these findings have been demonstrated in children with cystic fibrosis [8, 9].

PCD can result from defects in genes encoding the structural proteins of the ciliary components, for example, *DNAH5 *or *DNAI1*, which encode the ciliary dynein arm proteins. The search for novel genes responsible for the distinctive phenotypes of PCD is a hot topic in the current era of whole exome/genome sequencing projects [10, 11]. However, for the time being, most children have remained without recognized genetic defect [12].

Our phenotypic study aimed to analyze statural growth in a cohort of clinically diagnosed children and young adults with primary ciliary dyskinesia. We hypothesized that, similar to cystic fibrosis, PCD would affect the growth rate of affected individuals. We took advantage of the Czech pediatric system of regular preventative examinations of all children at their registering general pediatricians, which allowed for the retrospective collection of growth data for each child at 2-year intervals starting on the 1st birthday and ending at the age of 17 years old.

## 2. Patients and Methods

We analyzed retrospective growth data for 29 children and young adults (13 male subjects and 16 female subjects; 4 sibling pairs) diagnosed with primary ciliary dyskinesia (PCD). Their ages at the diagnosis of PCD ranged from 0.5 to 17 years old (median, 8 years), and their ages at the time of this analysis ranged from 1.5 to 24 years old (median, 14.5 years).

The diagnosis of PCD was established in accordance with the current recommended diagnostic protocol [13]. The diagnostic criteria were as follows:

1. clinical findings consistent with a diagnosis of PCD (otosinopulmonary symptoms);
2. abnormal video/high-speed video microscopy of cilia (immotile cilia or abnormal ciliary beat frequency and/or ciliary beat pattern) confirmed at least 2 times with an interval between examinations of at least 4–6 weeks; and
3. transmission electron microscopic findings consistent with a ciliary movement abnormality.

Sequencing of *DNAH5* and *DNAI1* was performed in patients with dynein arm defects, as described previously [14].

Of the 29 patients included in the analysis, situs viscerum inversus was present in 11 patients, bronchiectases in 21 patients, and hypoacusis and/or chronic secretory otitis in 14 patients. Causative gene defects in PCD-related genes had been identified in 10 of the 29 patients so far. In 9 patients, we identified homozygous or compound heterozygous pathogenic mutations in *DNAH5*, and in 1 girl, we identified compound heterozygous pathogenic mutations in *DNAI1* [14].

We collected all of the available measurements of statural height/length and body weight from the medical records of the affected individuals, acquired from their general pediatricians. Body height was measured using a wall-mounted stadiometer according to routine medical practice, and body length in children aged 1 year old was measured using a bodymeter. The data were collected at the time of regular preventative examinations, which were performed at the ages of 1, 3, 5, 7, 9, 11, 13, 15, and 17 years old. In total, 178 measurements of body height/length and weight were available for further analysis (median, 6 from each patient, range 1–9).

The data were converted into a standard deviation score (SDS) of body height, according to the latest available normative data of the background population [15, 16].

The mean body height and body mass index SDS values from all 29 patients at all time points were tested against the expected value of 0 using a 1-sample *t*-test. A *P* value < 0.05 was regarded as significant. In addition, we tested subgroups of patients with regard to (1) known genetic defects; (2) clinical recognition of PCD early or late in patient's life; (3) diagnosis of PCD within the last 10 years or earlier; and (4) selected clinical signs and symptoms, that is, the presence of situs viscerum inversus, severe ventilation defects, or bronchiectases and/or hypoacusis/chronic otitis media with effusion.

All of the subjects and/or their legal guardians provided informed consent for the genetic testing and for the analysis of their clinical data for research purposes.

## 3. Results

**Figure 1:**
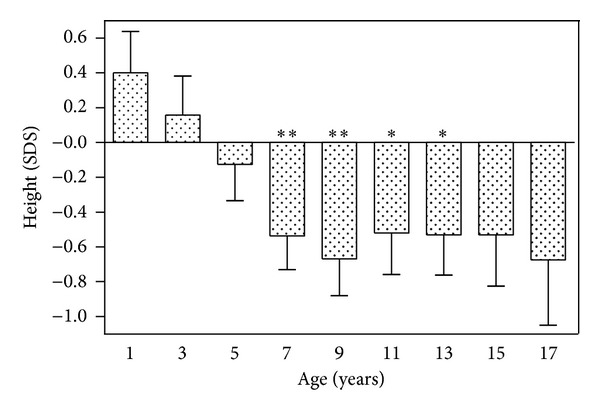
Body length/height (expressed as height SDS ± SEM) in 29 children with primary ciliary dyskinesia from their 1st through 17th birthdays, displaying the loss of height during childhood. *P < 0.05 and **P < 0.01 versus the expected value of 0 SDS.

Patients diagnosed with PCD progressively lost height during childhood, compared with their unaffected peers. Their body heights declined from +0.40 ± 0.24 SDS (mean ± SEM) on their 1st birthday, +0.16 ± 0.23 SDS at 3 years old, and −0.13 ± 0.21 SDS at 5 years old to −0.54 ± 0.19 SDS (*P* = 0.01 versus 0) at 7 years old, −0.67 ± 0.21 SDS (*P* = 0.005 versus 0) at 9 years old, −0.52 ± 0.24 SDS (*P* = 0.04 versus 0) at 11 years old, and −0.53 ± 0.23 SDS (*P* = 0.03 versus 0) at 13 years old. The progressive decline of body height was clearly expressing a decreased growth rate. After the age of 9 years old, the heights of the children with PCD stabilized. Only 12 patients reached the age of 17 years, and their heights (−0.67 ± 0.38 SDS) did not differ significantly from the background population (Figure 1 and Table 1).

**Figure 2:**
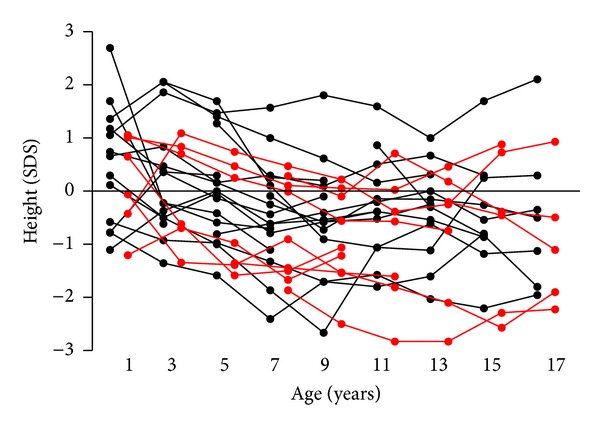
Individual length/height data (expressed as length/height SDS) obtained from the GPs' records of 29 children with primary ciliary dyskinesia. In red are patients with pathogenic mutations on both alleles of *DNAH5* or *DNAI1*; in black are all other patients with a clinical diagnosis of PCD.

No differences in body height were detected between male and female subjects diagnosed with PCD early and late during their childhoods, between those diagnosed within the last 10 years or earlier or between subjects with and without situs viscerum inversus, bronchiectases, impaired lung function, or hypoacusis/chronic otitis media with effusion. Individuals with known biallelic genetic defects in *DNAH5* or *DNAI1* had more pronounced growth retardation (Figure 2 and Table 1).

The body mass indices of children with PCD did not differ from the population standards, suggesting that a nutritional deficit was not the causative agent of the growth delay (Table 1).

## 4. Discussion

Multiple chronic conditions have been shown to compromise statural growth during childhood due to chronic wasting. In addition to malnutrition and chronic infections, which represent the most prevalent causes of acquired short stature worldwide, the significant nonendocrine causes of low growth rates in developed countries include inflammatory bowel disease, celiac disease, chronic renal failure, and chronic pulmonary disease [17–20]. In cystic fibrosis, growth failure can result from a combination of nutritional and pulmonary factors and from chronic inflammation.

The symptoms of PCD were described several decades ago and were originally defined clinically by Kartagener's triad. This triad consists of situs viscerum inversus, bronchiectases, and chronic sinusitis. Only later, this triad was found to be caused by immotile cilia [21]. Within the last decade, PCD has become an area of major research and clinical interest due to novel genetic findings, new diagnostic techniques, and recognition of the need for long-term therapeutic efforts that in part resemble the well-recognized efforts for individuals with cystic fibrosis [22]. The current concept of PCD largely exceeds the limits of Kartagener's triad.

PCD is associated with chronic diffuse pulmonary disease, with the gradual development of bronchiectases [4]. As evaluated by HRCT score, lung disease in these patients worsens early in childhood, although their spirometry can remain relatively stable during this period [23]. Lung function deterioration is usually observed in adulthood [7]. Fifty-two percent of PCD patients were reported to have obstructive sleep apnea syndrome [24]. PCD undoubtedly increases the risk of chronic deprivation, starting in many affected children during the neonatal period with chronic rhinitis and resulting in chronic pulmonary disease in many affected patients [22]. Therefore, it was logical to study the complex impact of PCD-related deprivation on body growth.

We have clearly demonstrated that PCD-related wasting negatively impacts growth rates during the childhood growth period, resulting in a mean loss of body height of approximately 0.5–1 standard deviations. According to clinical experience, the chronic/recurrent respiratory infections in patients with PCD are more frequent during preschool and early school years than thereafter. We can speculate that growth failure occurring during childhood may directly reflect the frequency and severity of infections. The limited number of affected individuals in our study did not allow us to clarify any potential links between the age at diagnosis and the severity of growth failure at this time. All of our PCD patients had shown signs of chronic mucopurulent/purulent bronchitis by the age of diagnosis. It can be assumed that earlier diagnosis of the disease and careful adherence to a recommended treatment regimen could result in a more favorable course for the disease, similar to the experience of children with cystic fibrosis [25].

In conclusion, we found a significant decrease in body height during childhood in individuals with PCD. Although we could not demonstrate the effects of early therapeutic interventions, we propose that early recognition of PCD, the introduction of effective therapy, and especially good compliance with recommended treatment regimens might diminish PCD-related wasting and improve the general outcomes of affected individuals.

## Figures and Tables

**Table 1:** Longitudinal data on body length/height and body mass index in a cohort of 29 children with PCD and in a subgroup of 10 children with known biallelic mutations in *DNAH5 *and *DNAI1*. The data are expressed as SDS ± SEM; the numbers of subjects with data available are shown in parentheses.

Age (years)	1	3	5	7	9	11	13	15	17
Length/height: all subjects (*n*)	0.40 ± 0.24 (19)	0.16 ± 0.23 (21)	−0.13 ± 0.21 (23)	−0.54 ± 0.19** (25)	−0.67 ± 0.21** (23)	−0.52 ± 0.24* (20)	−0.53 ± 0.23* (19)	−0.53 ± 0.29 (16)	−0.67 ± 0.38 (12)
Length/height: mutation carriers (*n*)	0.17 ± 0.36 (6)	0.00 ± 0.41 (6)	−0.54 ± 0.38 (7)	−0.72 ± 0.31* (9)	−0.91 ± 0.30* (9)	−0.92 ± 0.46 (7)	−0.78 ± 0.46 (7)	−0.68 ± 0.60 (6)	−0.96 ± 0.56 (5)
BMI: all subjects (*n*)	−0.29 ± 0.25 (16)	−0.42 ± 0.33 (18)	−0.02 ± 0.24 (20)	−0.26 ± 0.20 (22)	−0.30 ± 0.18 (20)	−0.10 ± 0.21 (18)	−0.25 ± 0.16 (19)	−0.21 ± 0.29 (16)	−0.38 ± 0.23 (12)
BMI: mutation carriers (*n*)	−0.71 ± 0.54 (5)	−0.51 ± 0.93 (5)	−0.11 ± 0.60 (5)	−0.32 ± 0.33 (7)	−0.37 ± 0.31 (7)	−0.10 ± 0.26 (6)	−0.29 ± 0.22 (7)	−0.50 ± 0.32 (6)	−0.51 ± 0.34 (5)

*P < 0.05 and **P < 0.01 versus the expected value of 0 SDS.
